# Discriminative machine learning for maximal representative subsampling

**DOI:** 10.1038/s41598-023-48177-3

**Published:** 2023-11-27

**Authors:** Tony Hauptmann, Sophie Fellenz, Laksan Nathan, Oliver Tüscher, Stefan Kramer

**Affiliations:** 1 Institute of Computer Science, Johannes Gutenberg University Mainz, Mainz, Germany; 2 The Leibniz Institute for Resilience Research, Mainz, Germany; 3 Department of Psychiatry and Psychotherapy, University Medical Center Mainz, Mainz, Germany

**Keywords:** Human behaviour, Computer science

## Abstract

Biased population samples pose a prevalent problem in the social sciences. Therefore, we present two novel methods that are based on positive-unlabeled learning to mitigate bias. Both methods leverage auxiliary information from a representative data set and train machine learning classifiers to determine the sample weights. The first method, named maximum representative subsampling (MRS), uses a classifier to iteratively remove instances, by assigning a sample weight of 0, from the biased data set until it aligns with the representative one. The second method is a variant of MRS – Soft-MRS – that iteratively adapts sample weights instead of removing samples completely. To assess the effectiveness of our approach, we induced artificial bias in a public census data set and examined the corrected estimates. We compare the performance of our methods against existing techniques, evaluating the ability of sample weights created with Soft-MRS or MRS to minimize differences and improve downstream classification tasks. Lastly, we demonstrate the applicability of the proposed methods in a real-world study of resilience research, exploring the influence of resilience on voting behavior. Through our work, we address the issue of bias in social science, amongst others, and provide a versatile methodology for bias reduction based on machine learning. Based on our experiments, we recommend to use MRS for downstream classification tasks and Soft-MRS for downstream tasks where the relative bias of the dependent variable is relevant.

## Introduction

A frequent challenge in social sciences and epidemiology is that a study sample is not representative for the entire population. Studies based on biased samples are not suitable for drawing conclusions because they lead to biased inferences about social processes^1^. Every phase of a research project, e.g. study design, data collection, or data analysis^2,3^ can induce bias, which is not dichotomous but can exist at various intensities. It is crucial for researchers to validate the presence of bias and in case of existence to mitigate is as much as possible to currectly analyze and interpret their findings.

Examples for bias are a survey that was conducted only in a specific city, although the goal was to generalize its results to the whole country or an online survey that depends on individual user participation, but people with a strong opinion on the topic have a higher likelihood to participate in the survey, leading to self-selection bias^4^. However, it is desirable to draw conclusions about all users or residents. If conclusions are drawn without bias correction, misleading descriptions of populations are produced and, ultimately, false conclusions are drawn^5^. Most bias cannot be completely removed without gathering more data, but bias reduction methods strive to mitigate bias as much as possible. Reducing bias can lead to more consistent findings and, therefore, saving time and resources, as fewer repeated studies are required^6^.

**Figure 1:**
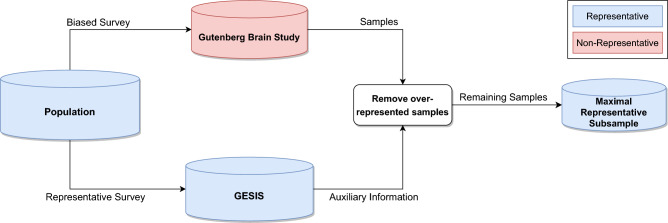
An overview of maximal representative subsampling. Both Gutenberg Brain Study and GESIS are surveys conducted on the German population, but contrary to GBS, GESIS was conducted representative. The auxiliary information of GESIS is linked to GBS with the help of a machine learning algorithm to detect and mitigate bias. The algorithm outputs a subsample with a reduced bias, which we call the *maximal representative subsample*. The data set visualizations differ in size to represent the number of samples they contain.

This article proposes a bias reduction method based on *positive and unlabeled training* (PU learning^7^) in combination with auxiliary information from population data. It takes advantage of available representative population data sets, which have been rigorously collected to prevent potential bias, representing the target population with high precision. We call our method *maximal representative subsampling* (MRS), because the representative distribution is used to remove elements from the biased data set to create a *maximal representative subsample*, which resembles the representative distribution. Figure 1 shows the structure of our method.

In more detail, the central aspect of MRS is to train random forests^8^ to differentiate instances from the biased data set and the representative data set. For each instance in the biased dataset, the trained classifier predicts the probability of that instance belonging to either of the two datasets. The predicted probabilities are used to choose which elements should be removed from the biased data set to make both data sets indistinguishable. Elements with a high probability, thus being overrepresented or significantly different, are removed. Training, sampling, and removal are repeated until a classifier cannot differentiate the data sets anymore. If the classifier is capable enough, it can be assumed that the generated subsample is now representative. As the aim of the generated subsample is to evaluate a research question to a required level of significance, it is necessary to keep the removal of instances at a minimum because pruning the data increases the uncertainty and lowers the statistical power, decreasing the insights of downstream analyses. This is especially harmful if the initial data set was already small.

Our methods make not only both data sets indistinguishable for a classifier but also decreases the *maximum mean discrepancy*^9^ (MMD) between the distributions. Furthermore, variables not contained in the representative data set and, therefore, not used during training, were shown to tend to approach the representative ratio as well if training variables and dependent variables correlate with each other.

The typical use case for MRS arises when the data acquisition has concluded and bias has been identified too late to support additional data collection efforts. In this situation, researchers would begin by obtaining a representative data set that accurately reflects the study population. Biased and representative data sets should overlap significantly in terms of variables to mitigate bias and prevent the introduction of artificial bias. In the optimal case, the representative data set encompasses the independent variable, thereby increasing the likelihood of its distribution will aligning with the representative one. Potential sources for acquiring such representative data sets include data archives, censuses, and market research institutes specializing in opinion analyses.

We tested the effectiveness of MRS on the *Gutenberg Brain Study* (GBS), a population-based study. The main aspect of the study was to measure how resilience influences voting behavior. Participants were randomly chosen and invited to participate in the survey. A downside of the survey is that it was carried out only in one city, which is not representative of the demographic of the country. Both are possible sources of selection and self-selection bias. Additionally, representative data of the German population were accessed from the Leibniz Institute for the Social Sciences and the Allensbach Institute for Public Opinion Research (Allensbach). The representative data sets were employed to reduce the bias of GBS with MRS in two separate experiments. Subsequently, further experiments were conducted on US Census Income^10^, which is assumed to be representative of the population of the United States, to illustrate how MRS deals with larger data sets. Artificial bias was induced, which allows us to gain more insight into the debiasing process.

Our paper is structured as follows: First, we give a detailed overview of the proposed method followed by the result discussion. After that, we reduce the bias of GBS twice with the information of two different representative data sets and analyze the changes over time. Afterward, to test long-term behavior and influence on a variable that is not included in the representative data set, we use a large public census data set, in which we introduced artificial bias. We compare MRS with other bias reduction methods that generate sample weights with respect to the quality of bias reduction and the usage of sample weights for downstream tasks. The paper ends with an outline of applying MRS to a statistical task.

In summary, the present article contains the following contributions:

- We propose two new approaches – MRS and Soft-MRS – to reduce selection bias.
- We develop a machine learning method to increase the representativeness of a data set by iteratively adapting weights, using the auxiliary information of a representative reference data set.
- We validate the application of sample weights to downstream classification tasks.
- We demonstrate how to use the representative subsample to fit a statistical model.

## Related work

Bias reduction algorithms exist in a great variety. Most of them rely on different types of additional auxiliary information, such as class probabilities^11^ or computing sampling distributions and using them to make appropriate corrections^12,13^ Techniques, such as cell weighting^14^ and raking^15^, use auxiliary information to compute sample weights and are used mainly to reduce non-response and non-coverage bias^16^.

In propensity score adjustment (PSA)^17^, a classifier is trained to estimate the participation propensities for individuals in the non-representative data set. The most commonly used method for PSA is logistic regression, but recent research showed that different machine learning methods can be advantageous in estimating the propensity score. At this point, it is not yet clear which algorithm is preferred for different distributions^18–20^.

As class distributions are rarely known and distribution estimation is a complex task in high-dimensional spaces, recent methods perform a distribution matching between training and testing sets, without a density estimation. *Kernel Mean Matching*^21^ (KMM) is a non-parametric method which directly produces sampling weights without distribution estimation by reweighting the samples so that the means of the training and test samples in a reproducing kernel Hilbert space are close. The authors showed that KMM improves learning performance compared to training on unweighted data and, in some cases, can even be better than the true sample bias distribution.

Another method is the *Kullback-Leibler Importance Estimation Procedure*^22^ (KLIEP), which also directly estimates the importance of the sample without density estimation and implements a natural model selection procedure. KLIEP finds an importance estimate such that the Kullback-Leibler divergence from the true test input density to its estimate is minimized. Its key advantage over KMM is that the training samples are not cross-validated, but the test input samples. This allows the accuracy of model selection to be improved, as the number of training samples is typically limited while the test input samples are readily available^22^.

Existing methods estimate sample weights with machine learning algorithms or iteratively optimize sample weights, but none combines a discriminative machine learning method with iterative refinements. That is why we developed with MRS a method that matches distributions without an explicit density estimation with a machine learning method that allows it to model complex dependencies with an approach that can fine-tune sample weights iteratively.

## Methodology

### Maximal representative subsampling

This section describes in more detail the components of MRS. MRS is based on PU learning^7^, which trains a semi-supervised binary classifier with only positive and unlabeled data. It is used for tasks such as matrix completion and multi-view learning. Furthermore, it can classify data streams and time series and detect events, like co-occurrences, in graphs^23,24^. PU learning requires a positive set *P* and a mixed set *U*, which contains positive and negative samples. Its purpose is to train a classifier that can classify a test set *T*, in which the labels are unknown, into positive and negative samples. During training all elements in *U* are treated as elements of the negative class. We adapt PU learning to our experiments using the representative data set as *P* and denoting one part of the biased data set as an unlabeled set *U* and the remaining samples as *T*. The classifier assigns the elements $t \in T$ a probability value of being an element of the representative or non-representative data set. Probabilities are used to remove elements from the biased data set until a specified stopping criterion is fulfilled. When MRS stops, the current sub-sample, which is assumed to be representative, is returned.

To confirm that MRS not only makes data sets indistinguishable but also increases the distributions’ similarity, MMD, which measures the distance between two distributions, is used. MMD can be computed as the norm of the difference between the distributions’ feature means in the reproducing kernel Hilbert space^25^.

Given two sets of samples $X= \{x_i\}_{i=1}^n$ and $Y=\{y_i\}_{i=1}^m$, one can compute the empirical estimate of the MMD in the following way:

1 $$\begin{aligned} \begin{aligned} \text {MMD}(X, Y) = \left[ \frac{1}{m^2}\sum _{i,j=1}^{m} K(x_i, x_j) - \frac{2}{mn} \sum _{i,j=1}^{m,n} K(x_i, y_j) + \frac{1}{n^2} \sum _{i,j=1}^{n}K(y_i, y_j)\right] ^{\frac{1}{2}}, \end{aligned} \end{aligned}$$

where *K* is the radial basis function kernel:

2 $$\begin{aligned} \begin{aligned} K(x, y) = e^{(- \frac{\Vert x-y\Vert ^2)}{2\sigma ^2}}. \end{aligned} \end{aligned}$$

Similarly to Gretton et al.^9^, we determine $\sigma$ heuristically, setting it to the mean distance between the samples in the aggregated sample, which contains all samples from both data sets.

MRS’ pseudocode is given in Algorithm 1. The inputs are the representative data set, the non-representative data set and the number of elements that get dropped at every iteration. The number of elements dropped every iteration is a trade-off between accuracy and computing time. The more samples are dropped per iteration, the faster the method is because it reduces the number of iterations, but dropping more samples results in fewer retrained classifiers, thus, potentially reducing the accuracy. The runtime furthermore depends on the size of the representative data set, the size of the non-representative data set, and the type of classifier.

In each iteration of the outer loop (lines 2–13), *k* instances are removed from the biased data set *N* until the classifier cannot distinguish *R* and *N*, which is measured by *Area Under the Receiver Operating Characteristic Curve* (AUROC) between *R* and the current *N*. The algorithm stops if $|\text {AUROC}-0.5| \le \delta$ with $\delta =0.001$ in our experiments. Before each iteration classifier is trained to calculate the current AUROC and to test the stopping criterion.

**Algorithm 1:**
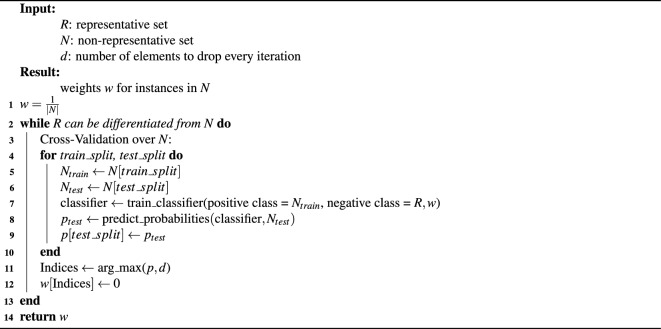
Maximal representative subsampling.

Optimizing hyperparameter for MRS requires either to run it completely with different configurations or to perform a nested cross-validation in each iteration. Both approaches make the method more computational intensive, which would lower its acceptance. Soft-MRS on the other hand does not use a cross-validation and does not need to train an additional classifier to test the stopping criterion, which requires only one optimization per iteration.

To determine which instances will be removed, we obtain a probability for each instance with a 5-fold stratified cross-validation (lines 3–10). In every round of the cross-validation, a classifier is optimized and trained on both data sets to predict the probabilities on the hold-out set (lines 7–8). Probabilities are aggregated over the hold-out sets (line 9) and returned.

MRS can use any classifier that predicts probabilities, and we selected random forests^8^, because they are intuitive and fast to train on standard hardware. The trained random forest predicts probabilities of belonging to either the positive or negative class for the holdout set.

The weights of the *k* elements (line 11) with the highest probabilities are set to 0 to remove them (line 12). After removing them, the new AUROC is computed to verify the stopping criterion. In our experiments, the stopping criterion was the AUROC of a decision tree with minimal cost-complexity pruning^26^. If the stopping criterion is met, the current sample weights vector *w* is returned, otherwise, the algorithm continues with the next iteration.

An ablation study for MRS is included in the Supplementary Material.

### Soft maximal representative subsampling

Removing samples with MRS can be too restrictive for smaller data sets, potentially reducing its size too much, undermining further analyses. In response to this concern, we developed a “soft” variant of MRS (Soft-MRS). Instead of discarding samples entirely (i.e. setting weights to zero), Soft-MRS takes a different approach by calculating and adjusting sample weights.

Soft-MRS (Algorithm 2) starts by first setting the sample weights to a uniform distribution (line 1). After that, sample weights are iteratively adjusted until the data sets are indistinguishable (outlined in lines 3–16). In our experiments, we used MMD to measure the difference between data sets and to validate the stopping criterion. The update loop continues until the MMD no longer decreases, employing a patience of 25 iterations. The objective is to adjust the sample weights in order to minimize the distance between the data sets.

**Algorithm 2:**
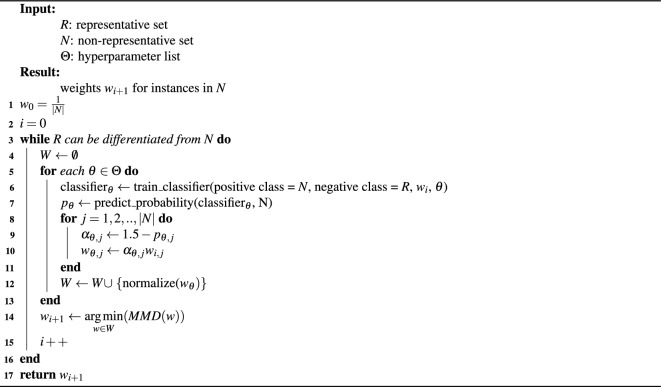
Soft maximal representative subsampling.

As the weights of the same instances are updated by a multiplicative update (see line 10 in the pseudocode and the explanation below), in a style reminiscent of AdaBoost^27^ or additive regression^28^, over and over again, the classifier itself can be kept very simple compared to MRS. Effectively, a single unpruned decision tree (inhibited in growth only by a minimum weight fraction per leaf) per iteration is required to make the scheme work. This makes the training of classifiers by a factor of $k \times l$, *k* being the number of test sets in the cross-validation and *l* being the number of trees in the random forest, faster than the training of a classifier in MRS. Additionally, one saves time by the MMD stopping criterion versus the cross-validated cost-complexity pruned tree in MRS. The savings in running times can then be invested in the optimization of hyperparameters, which is shown in lines 5-13 in the pseudocode.

Therefore, the following steps are performed in each iteration to update the weights (lines 5–14): For each hyperparameter configuration, a decision tree classifier is trained to distinguish the representative and non-representative data set to predict probabilities for *N* (lines 6–7). The weight update $\alpha _i$ for a sample *i* depends on its predicted probability $p_i$ and is calculated by the linear function $1.5 - p_i$ (lines 9–10). The aim is that samples which are predicted with a high confidence to belong to the representative sample obtain a higher sample weight and the ones with a high confidence of belonging to the non-representative samples a lower sample weight. A predicted probability of 0 decreases the weight by 50%, contrary to a prediction of 1, which increases it by 50%. The weight changes are bigger if the predicted probability deviates further from 0.5 and they decrease as the difference gets smaller, approaching 1 and so keeping the weight constant.

The updated weights are selected greedily, choosing the set of sample weights that leads to the lowest MMD value (line 14). Once the stopping criterion is met, the weights from the current iteration are applied to the downstream task (line 17).

## Discussion

Deriving knowledge from biased data sets is problematic, hence, it is desirable to reduce potential bias sources in the gathering process, but sometimes researchers detect bias too late, when it is already impossible to gather additional data. In this case the preferred course of action is to employ a bias mitigation method. Existing debiasing methods are based either on successive optimization procedures or discriminative models, but no method combines both paradigms.

Addressing this gap, we developed two methods, MRS and Soft-MRS, which iteratively adapt sample weights through machine learning. MRS employs PU learning to remove discriminating samples, while Soft-MRS performs multiplicative updates on all sample weights, providing an alternative approach to bias reduction. The iterative adaptation of weights in both methods contributes to the creation of unbiased and representative samples from initially biased datasets. The benefits of the methods are as follows:

### Uniform sample weights

MRS introduces the advantage of assigning equal weights to all remaining samples. This equal contribution simplifies subsample interpretation and mitigates the risk of overemphasizing samples from underrepresented groups. In contrast to weighting methods, where samples receive very low weights but are still used, MRS ensures a reduced effective sample size, potentially benefiting downstream tasks (in terms of running times or interpretability, e.g. in case of instance-based methods). It has also the advantage that the resulting sample can directly be used as input for algorithms that cannot incorporate sample weights without an additional sampling step.

### Bias reduction

Soft-MRS achieved the lowest mean rank in terms of MMD and relative bias reduction. The reduction for MRS, reliant on the AUROC stopping criterion, could further be improved by adapting it to use MMD. However, this adjustment would shrink the dataset, diminishing statistical power, as AUROC stagnates around 0.5 before MMD stops decreasing, necessitating the removal of more samples.

### Improvement of downstream classification

Although statistical significance may not always be observed, the application of MRS in downstream classification consistently shows competitive results in most cases, underscoring its potential to enhance classification tasks on biased datasets.

### Adaptability

Whereas the previous points concern more the end users of the methods, the final point concerns method developers who wish to adjust them or develop them further: MRS and Soft-MRS provide versatile, modular, machine-learning based frameworks for bias reduction, allowing adaptation to various use cases. Potential adjustments include selecting different classifiers or modifying the stopping criterion. For MRS, one could explore alternative methods to determine which samples to remove, and for Soft-MRS, alternatives to the weight update function. This adaptability to specific use cases renders both variants well-suited for a wide range of tasks. However, they can also inspire further research involving different module combinations and downstream tasks.

In conclusion, the introduced methods, MRS and Soft-MRS, present valuable tools for researchers and practitioners addressing bias in their data sets. Their adaptability, demonstrated effectiveness in bias reduction, and comparability to existing methods position them as versatile approaches for a wide range of applications. Ongoing exploration of optimal use cases and continuous experimentation will further refine and extend the utility of these methods in tackling bias in real-world scenarios.

## Results

### Statistical correction of Gutenberg brain study

In our first experiment, we performed a bias statistical correction of the Gutenberg Brain Study (GBS, n = 579). GBS is a population-based sample of healthy adults aged 18 to 75 years who live in a city located in southwest Germany, Mainz. Its main research questions were resilience and its influence on political participation.

The acquisition of the study began with a random selection of potential participants through an official local resident register. Based on this information, an invitation letter to the study was sent to potential participants. Participants participated in a telephone survey. To determine the resilience of the participants, a German version^29^ of the *brief resilience scale* (BRS) was used. BRS consists of six different questions, and resilience is considered a positive adaptation to past and ongoing exposure to potential adverse effects of stressors^30^. The questions are rated on a five-point Likert scale^31^, although half of the questions are negatively formulated and half positively. To evaluate the questionnaire, they reversed the coding of the negatively formulated items to calculate the mean of the six items^32^. Additionally, participants were asked about their willingness to participate in an upcoming political election at the time of the survey. Participants were contacted again after the election to inquire if they had actually participated. The survey aimed to investigate a potential correlation between resilience and political participation. However, people with greater resilience or a high interest in politics could have been more willing to participate, leading to self-selection bias. Additionally, all the people were residents of the same city, where a university is located. Therefore, its population contains an increased percentage of residents with higher education compared to the rest of the country. This is why the survey data overrepresent higher income and higher education groups, as the participants were selected primarily from an academic environment. Therefore, the conclusions drawn cannot be generalized to the entire population of Germany.

We used the auxiliary information of two representative data sets to perform different statistical corrections of GBS. In this way, we can validate that MRS works with different data sets. The first representative data set originates from the Data Archives for the Social Sciences, maintained by GESIS - Leibniz Institute for the Social Sciences, which holds comparable and representative studies in politics and psychology. The acquired data set (GESIS, n = 4249, n = 3869 after removing participants with missing values) includes the German-speaking population with permanent residence in Germany.

GESIS provided the auxiliary information to perform the statistical correction on GBS. The algorithm was repeated ten times and the mean and standard deviation of the metrics were calculated on the runs. A sample was removed from GBS in each iteration and the maximal representative subsamples contained between 119 and 171 remaining samples.

**Figure 2:**
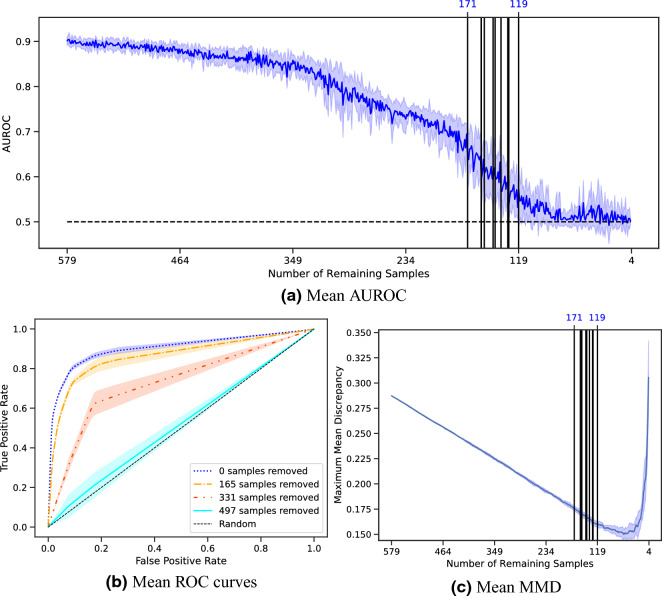
AUROC, ROC curve, and MMD of the statistical correction of GBS with auxiliary information from GESIS. An element is dropped per iteration and the algorithm is run until no samples remain. The experiment was repeated ten times. The dark lines represent the means, and the surrounding shades visualize the standard deviations. The vertical lines indicate the iteration in which the remaining samples were declared to be maximal representative subsamples, and the top number represents the number of remaining samples. The ROC curve is given in four different steps.

Figure 2 gives an overview of how the metrics changed during the execution of MRS. AUROC decreases constantly over time with only a few exceptions (Fig. 2a). The vertical black lines mark the iterations at which the remaining samples were chosen to be the maximal representing subsample. Additionally, the ROC curves at the beginning and every time 165 samples were removed (Fig. 2b) are visualized. The ROC curves converge to the random line and lie almost on it after 497 samples were removed. The progression of MMD confirms a constant decrease in the distribution distance of both data sets, except in the end, when the biased data set becomes too small for a reliable comparison (Fig. 2c).

In our second experiment, we show that the general results of MRS are robust to the choice of a representative data set. Small changes in the results are expected due to the different intersections of variables and the distribution of the representative data set.

Auxiliary information was used from another representative data set from the Allensbach Institute for Public Opinion Research. The cross-sectional data set was collected between August and September 2016. Normalized face-to-face interviews were conducted nationwide with 1128 adults 18 years and older. The interviewers were trained and could ask questions in the event of uncertainty. Based on official German statistics (microcensus in 2015^33^), individuals were selected if they met the criteria for a quota sample with respect to age, sex, education, professional position, region, and city size. After removing samples with missing data, 1082 samples remained. Again, ten MRS iterations were performed with a drop rate of one sample.

**Figure 3:**
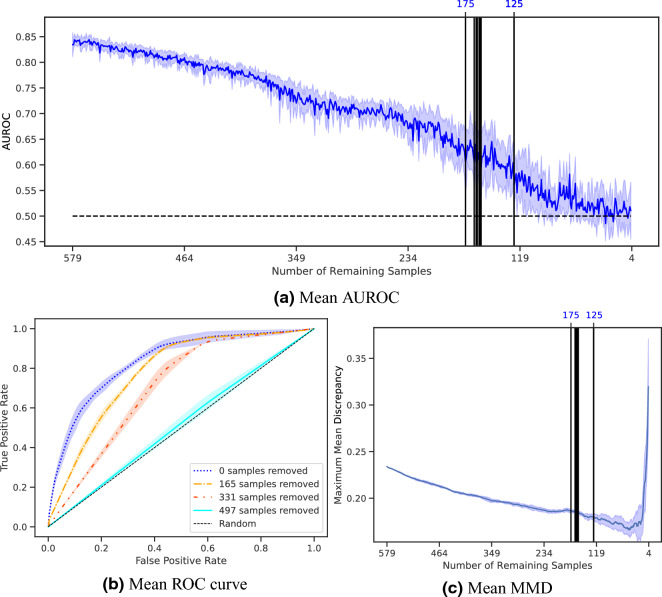
AUROC, ROC curve, and MMD of the statistical correction of GBS with Allensbach’s auxiliary information. Five samples are dropped per iteration, and the algorithm is run until no samples remain. The experiment was repeated ten times. The dark lines represent the means, and the surrounding shades visualize the standard deviations. The vertical lines indicate the iteration in which the remaining samples were declared maximal representative subsamples and the number on the top represents their number of samples. The ROC curve is given every time 165 samples were removed.

Figure 3 shows the results of the statistical correction of GBS with Allensbach’s auxiliary information. The general decreasing trend of the metrics is the same, but minor changes occur due to the different distribution and intersection of the representative data set. The initial MMD of GBS and Allensbach is lower but the mean AUROC decreases slower. Nevertheless, MRS stops around the same iteration as shown in Fig. 3a and b. The maximal representing subsamples contain between 128 and 175 samples. The more samples are removed by the algorithm, the closer the ROC curve is to the diagonal. This indicates that the classifier can no longer distinguish the representative from the non-representative data set. Again, the MMD decreases with each iteration until the *N* gets too small, showing that the distance between the distributions decreases with the removal of the samples (Fig. 3c).

### US census income

The following experiment sought to understand how our method incorporates attributes that are not contained in the representative sample, and, therefore, are not visible during training. The assumption is that the unseen variables attain a representative distribution through correlation with the visible variables, allowing them to draw conclusions from the subsample. We used the US Census Income data set for 2018 for California loaded with Folktables^34^, as it contains a wide variety of demographic attributes and a large number of samples. The task for US Census Income is to predict if the income of a person is higher than fifty thousand per year. The biased variable income was omitted from the classifier training because it would reveal the representative ratio.

In every iteration we sampled a subsample of 5000 samples from US Census Income. The subsample was divided into two parts: one for the representative data, which provides auxiliary information for statistical correction. I the other one artificial bias is added and it is used as non-representative data set.

We performed three different experiments: In the first experiment, the fraction of the negative class was reduced and in the second experiment the fraction of the positive class was reduced. Only 10% of the samples of the bias class were kept and all samples for the other class. Both data sets are biased because they do not represent the state-wide income ratio. In the last experiment, both fractions were kept unchanged to create a second representative data set, which was used to examine the properties of MRS on an already representative data set.

In addition to the metrics previously used, the relative bias (Eq. 3) of the target in the non-representative data set was calculated to monitor whether it decreases:

3 $$\begin{aligned} \text {Relative Bias} = |\frac{\bar{Y}-\bar{y}}{\bar{Y}}| * 100, \end{aligned}$$

where $\bar{Y}$ is the representative mean of the target variable and $\bar{y}$ is the weighted mean of the non-representative data set.

**Figure 4:**
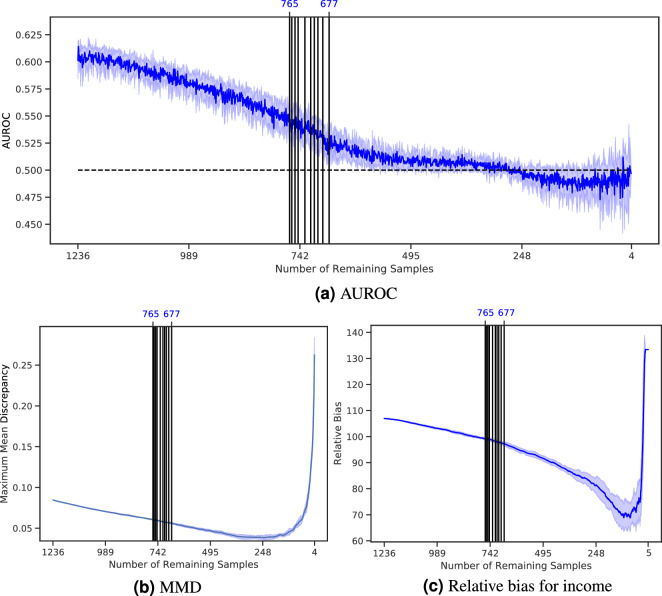
AUROC, ROC curve, and MMD of the statistical correction of the US Census Income subset containing less negative samples (low income) with the auxiliary information of the representative subset. The vertical lines indicate the iteration in which the remaining samples were declared to be maximal representative subsamples, and the top numbers represent remaining samples.

At first, MRS was employed to debias the sample that contained a decreased number of lower-income samples. The AUROC starts already with a low value (Fig. 4a), but gradually decreases and eventually reaches the random line. The MMD also continually decreases over time (Fig. 4b), showing that the algorithm reduces the distance between both distributions. The relative bias starts high and decreases over time until the data set becomes too small to be representative (Fig. 4c). It should be noted that at the time that MRS stopped and returned the maximum representative subsample, the relative bias and MMD did not had their minimum value.

Results for the remaining experiments are included in the Supplementary Material.

### Method comparison

To validate the applicability to real-world tasks, we tested MRS and Soft-MRS on five different data sets. The tasks represent use cases where only dependent variables for the non-representative sample are known, while simultaneously access to a representative sample with identical features, but without the dependent variable, is available. On the one hand we tested if the training on the weighted data set increases the predictive performance on the representative sample, on the other hand, the distances between data sets and the relative bias were validated.

We compared MRS, Soft-MRS, naive uniform weighting, KMM, and PSA. For KMM, we determined $\sigma$ again heuristically by computing the mean distance between samples in the aggregated set. PSA was used with logistic regression, where we calculated the sample weights with the inverse propensity score: $(1 - \pi ) / \pi$^35^.

The experiment was performed on five public data sets, which we are going to describe from the largest to the smallest one with regard to the number of samples: The first and biggest data set is US Census Employment, which is a classification task from Folktables, where the goal is to predict whether a person is employed. The second data set is US Census Income from Folktables. The next data set is Human Research Analytics, where the task is to predict whether a person wants to work for the company after training courses. For the fourth data set, Breast Cancer Wisconsin^36^, the task is to predict whether a sample, described by the characteristics of the digitized images, is malignant or benign. The last and the smallest data set is Loan for which the task is to predict whether a person is eligible for a home loan. The data set characteristics are given in the Supplementary Material.

We randomly split each data set into a training and a test set, each containing 50% of the samples. To create artificial bias in the training set, we used a sample selection mechanism that chooses samples less often when they are further away from the sample mean $\bar{x}$, i.e. $P(s_i|x_i)\propto \exp (-\sigma ||x_i-\bar{x}||^3)$, with $\sigma =-0.05$^21^. For US Census Employment and Income, we drew 5000 samples in every iteration before splitting, because the complete data set contains too many samples.

We then calculated the sample weights with the biased training set and the test set. Subsequently, a decision tree classifier with cost complexity pruning was trained on the weighted non-representative data set to predict the targets for the representative data set. The test results were derived from an average of 50 trials, and the AUROC and the *Area Under the Precision-Recall Curve* (AUPRC) were calculated using the predictions of the decision tree. In addition, we assessed MMD and the relative bias associated with the target variable. This section focuses on a discussion of AUROC and AUPRC (Table 1), while the remaining measures can be found in the Supplementary Material.

PSA achieved twice the second best AUROC and once the second best AUPRC, but improves them only for one data set, making the method impractical for downstream classification tasks.

KMM also does not perform well and reduces for four of five data sets the classification quality. A possible explanation for the weak results is that a weight distribution computed in a infinite-dimensional kernel space could be not suitable to train a decision tree, whereas the weights of MRS and Soft-MRS are calculated already using decision trees, which could be an advantage for downstream classification tasks.

MRS works best on the two smallest data sets, Human Research Analytic and Us Census Employment and increases in three cases the classification results. Overall, MRS is the best method in the mean ranking for AUROC and AUPRC. If we take only the larger data sets into account, MRS has again the best mean rank for AUROC and AUPRC. The removal of negative samples with MRS increases the classifier’s focus on positive samples and increases the probability to correctly classify them, which increases the AUPRC.

Soft-MRS only increases in one case the AUROC and in two cases the AUPRC, but achieved best results for MMD and relative bias as can be seen in the Supplementary Material.

**Table 1:** Downstream task AUROC and AUPRC over 50 iterations.

Metric	Method	US census income	US census employment	Human research analytic	Breast cancer	Loan	All mean rank	Large mean rank
AUROC	Uniform	$\mathbf {0.809}\pm \mathbf {0.013}$	$\mathbf {0.857}\pm \mathbf {0.014}$	$0.730\pm 0.014$	$\it 0.699 \pm 0.026$	$\it 0.942 \pm 0.021$	*2.3*	*2.50*
	PSA	$\it 0.807 \pm 0.016$	$0.850\pm 0.014$	$\it 0.731 \pm 0.013$	$0.690\pm 0.030$	$0.938\pm 0.019$	3.7	3.17
	KMM	$0.797\pm 0.012$	$0.845\pm 0.013$	$\it 0.731 \pm 0.013$	$0.662\pm 0.068$	$0.940\pm 0.023$	4.4	4.50
	MRS	$0.805\pm 0.017$	$\mathbf {0.857}\pm \mathbf {0.014}$	$\mathbf {0.732}\pm \mathbf {0.015}$	$\mathbf {0.700}\pm \mathbf {0.024}$	$\mathbf {0.945}\pm \mathbf {0.016}$	**1.6**	**2.00**
	Soft-MRS	$0.803\pm 0.016$	$\it 0.855 \pm 0.013$	$\mathbf {0.732}\pm \mathbf {0.014}$	$0.697\pm 0.029$	$0.940\pm 0.021$	3.0	2.83
AUPRC	Uniform	$\mathbf {0.726}\pm \mathbf {0.023}$	$\mathbf {0.766}\pm \mathbf {0.027}$	$0.391\pm 0.021$	$\it 0.790 \pm 0.022$	$0.951 \pm 0.019$	*2.6*	*2.50*
	PSA	$\it 0.723 \pm 0.024$	$0.757\pm 0.028$	$0.392\pm 0.022$	$0.787\pm 0.023$	$0.948\pm 0.017$	3.8	3.33
	KMM	$0.707\pm 0.022$	$0.748\pm 0.021$	$\it 0.393 \pm 0.021$	$0.772\pm 0.041$	$0.949\pm 0.020$	4.3	4.17
	MRS	$0.718\pm 0.024$	$\mathbf {0.766}\pm \mathbf {0.026}$	$\mathbf {0.394}\pm \mathbf {0.023}$	$\mathbf {0.791}\pm \mathbf {0.022}$	$\mathbf {0.954}\pm \mathbf {0.015}$	**1.6**	**1.83**
	Soft-MRS	$0.717\pm 0.025$	$0.763\pm 0.024$	$\it 0.393 \pm 0.021$	$\mathbf {0.791}\pm \mathbf {0.022}$	$\it 0.951 \pm 0.020$	2.7	3.17

The numbers are mean values and standard deviations. The mean rankings over the data sets were once computed for all data sets and once for the three data sets with more than 2000 samples (US Census Income, US Census Employment and Human Research Analytic). Best values are written bold and second best italic.

### Statistical analysis

In order to address the application of MRS, we applied it to a real-world scenario involving a psychological survey. The central aim of the survey was to investigate and evaluate the impact of varying levels of resilience on individuals’ participation in elections. For this purpose, MRS and Soft-MRS were employed to debias GBS, and, subsequently, logistic regression models were computed for the maximal representative subsample, the weighted sample and the uniformly weighted sample.

The null hypothesis is that there is no correlation between the BRS and participation in a election. A *p*-value $\le 0.05$ was chosen to reject the hypothesis. Because our dependent variable is binary, we performed a logistic regression with the BRS as independent variable and participation in the election as dependent variable. Additionally, MRS was applied on GBS with Allensbach’s auxiliary information to calculate the maximal representative subsample. Allensbach was used because it also contains the independent variable and could lead to a better representation of its distribution.

**Figure 5:**
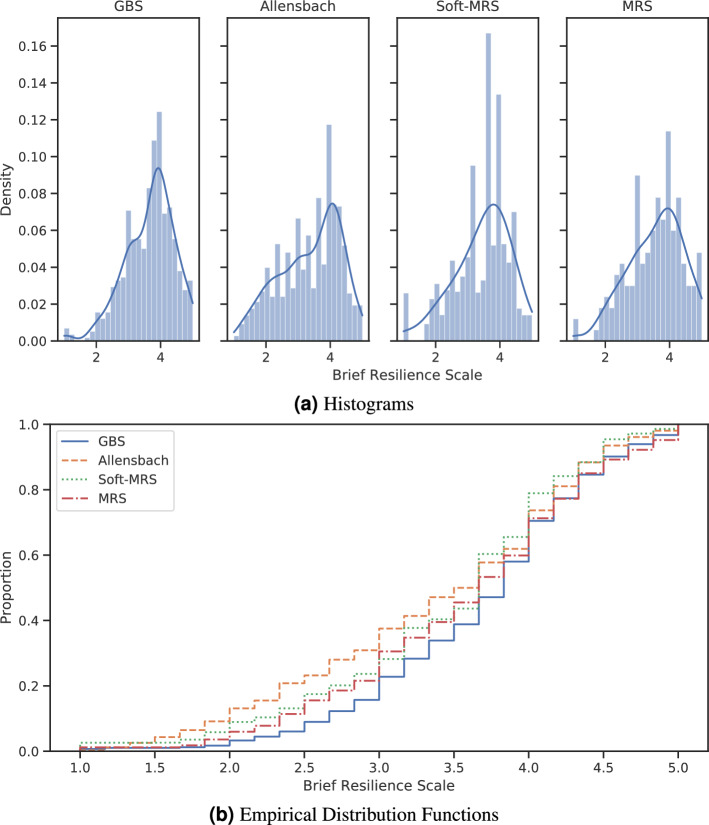
Comparison of the brief resilience scale of GBS, Allensbach, MRS, and Soft-MRS. (**a**) Histograms. (**b**) The cumulative distribution functions of the histograms.

Figure 5a presents the histograms of Allensbach, GBS, MRS, and Soft-MRS. Compared to the representative distribution of Allensbach, it is evident that GBS lacks low BRS values. The two histograms on the right are from MRS and Soft-MRS and show that the new distributions are closer to Allensbach’s. This is more apparent in the empirical distribution functions of the histograms (Fig. 5b).

The empirical distribution functions of MRS and Soft-MRS are located between those of Allensbach and GBS. Both figures show that the distributions of MRS and Soft-MRS approach that of Allensbach, but they also visualize one weakness of weighting methods; they cannot create additional samples. This is especially noticeable in the region between 1.0 and 2.0, where GBS has almost no samples, but Allensbach does have them, which cannot be corrected because MRS creates no artificial samples. Soft-MRS increases the weights in this area to compensate for it. Soft-MRS assigns samples with a higher-valued BRS a high weight to compensate for missing lower-valued samples, which leads to a gap of medium-valued samples.

To quantitatively measure the differences in BRS, we calculated the Wasserstein distance and relative bias of BRS between GBS, MRS and Soft-MRS, and Allensbach. The results were 0.337 and 9.031 for uniform weighting, 0.220 and 5.819 for MRS, and 0.173 and 2.477 for Soft-MRS. This shows that both methods increase the similarity to the representative BRS distribution.

Finally, we measured the correlation between BRS and voting behavior with a logistic regression for GBS, MRS, and Soft-MRS using the likelihood ratio test to calculate *p*-values. The *p*-value of GBS is 0.453, for MRS 0.562 and for Soft-MRS 0.624, making the survey representative changed the *p*-values, but there is insufficient evidence to reject the null hypothesis. This indicates that relying solely on BRS is not a suitable indicator of participation in an election, but it is important to acknowledge that the number of nonvoters and individuals with low BRS is already low in GBS, MRS compensates for this by removing persons with high BRS; however, removing elements from a sample typically results in decreased statistical power.

Participation in voting represents only one aspect of political participation. A subsequent research survey could explore the influence of resilience on various other types of political participation, such as running for office, affiliating with a political organization, or engaging in social activism.

### Supplementary Information

Supplementary Information.

## Data Availability

GBS, GESIS, and Allensbach are not publicly available. US Breast Cancer https://archive.ics.uci.edu/ml/datasets/breast+cancer+wisconsin+(diagnostic), Human Resource Analytic (https://www.kaggle.com/datasets/arashnic/hr-analytics-job-change-of-data-scientists) and Loan (https://www.kaggle.com/datasets/burak3ergun/loan-data-set) are freely available online. Folktables can be accessed through its Python package (https://github.com/socialfoundations/folktables).

## Code availability

The source code for the preprocessing of GBS, Gesis and Allensbach (https://github.com/laksannathan/maximal-representative-sampling) and for the methods and experiments (https://github.com/kramerlab/WeightDebiasing) is available on GitHub.
