# Supplementary material for: Discriminative machine learning for maximal representative subsampling
Source: Sci Rep. 2023 Nov 27;13:20925. doi: 10.1038/s41598-023-48177-3 (PMC10684887; doi:10.1038/s41598-023-48177-3)
Supplement: Supplementary file 1 — Supplementary Information. [file 41598_2023_48177_MOESM1_ESM.pdf]

Discriminative Machine Learning for Maximal Representative Subsampling

Tony Hauptmann[1, *], Sophie Fellenz[1], Laksan Nathan[1], Oliver Tüscher[2,3], and Stefan Kramer[1]

[1]Institute of Computer Science, Johannes Gutenberg University Mainz, Mainz, Germany
[2]The Leibniz Institute for Resilience Research, Mainz, Germany
[3]Department of Psychiatry and Psychotherapy, University Medical Center Mainz, Mainz, Germany
[*]Corresponding author: thauptmann@uni-mainz.de

A Ablation Study

An ablation study was carried out to better understand the individual components. In it, different components of the method were omitted, and changes were compared. We validated our experiments with the *area under the receiver operating characteristic curve* (AUROC) and the *maximum mean discrepancy* (MMD) as metrics. The AUROC ranges from 0 to 1, where values near 0.5 describe a method that uses random guesses, and values closer to 1 indicate an ideal classifier.

To additionally measure the distance between the distributions, we used MMD to verify that the subsample has a lower distance to the representative data set. Since MMD is a distance measurement, its range varies between 0 and ∞, and low values highlight similarities between the two sets.

Random drop

First, we tested MRS to see if it produces better results than the naive baseline without any probability estimation, where the samples are randomly dropped (Figure 1). The algorithm did not have any information of the representative data set and each element had the same probability of removal.

Compared to MRS, the AUROC of the random drop decreases at a much lower rate and reaches the random line in a later iteration (Figure 1a). Differences in MMD values (Figure 1b) further support our hypothesis that MRS decreases the differences in the distributions. The random drop MMD values never decrease and are constantly higher than the corresponding MRS values.

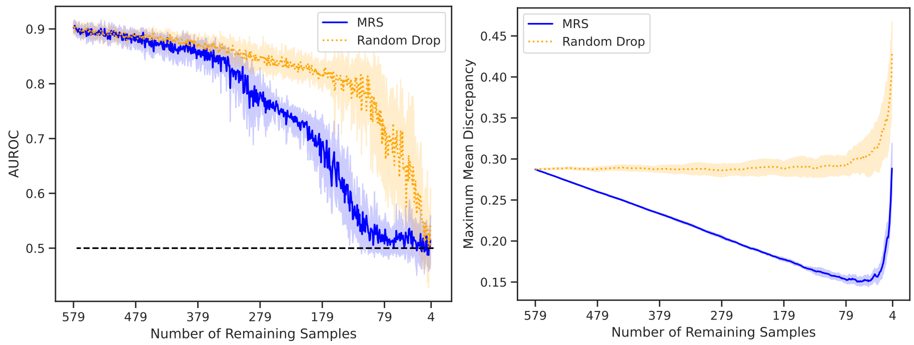

(a) AUROC (b) MMD

Supplementary Figure 1: AUROC and MMD of the statistical correction of GBS with the auxiliary information of GESIS. The MRS metrics and a random drop variant are compared. The experiment was repeated ten times. The dark lines represent the means, and the surrounding shades denote the standard deviations.

That assures that the method actively reduces the differences in the distribution and that the effects are not solely based on the reduced data set size.

Cross-Validation

In the second test, we investigated the influence of cross-validation by comparing MRS with a variant without cross-validation.

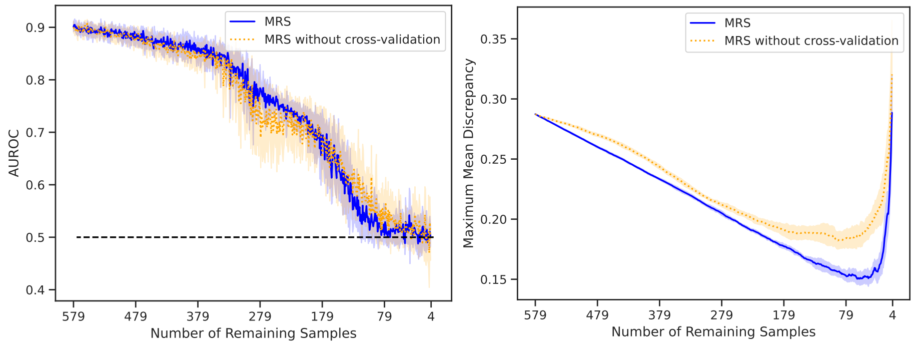

(a) AUROC (b) MMD

Supplementary Figure 2: AUROC and MMD of the statistical correction of GBS with the auxiliary information of GESIS. This figure compares the MRS metrics and a variant without cross-validation. The experiment was repeated ten times. The dark lines represent the means, and the surrounding shades denote the standard deviations.

As shown in Figure 2, the algorithm performs slightly better using cross-validation. This is not apparent in the AUROC values, as both perform simlar (Figure 2a), but it is more apparent in the comparison of the MMD values, where MRS performed slightly better overall (Figure 2b). We hypothesize that this is due to the relatively small size of GBS, because cross-validation ensures that all instances are chosen equally often and that every instance is guaranteed to be chosen for the training set, enabling more confident estimates.

B Further experiments on US Census Income

B.1 Less positive class

In contrast to the aforementioned experiment, the non-representative dataset contains fewer high-income samples. Figure 3 shows similar results to the experiment with an increased fraction of low-income samples. The AUROC decreases until it bounces around the 0.5 line (Figure 3a), the MMD and relative bias decrease until the data set is too small to maintain similarity and increases again (Figures 3b and 3c).

B.2 Representative

To examine how the method behaves on an unbiased dataset, we performed an experiment with two representative data sets (Figure 4). The AUROC in the first iterations was already small enough that MRS would have stopped after removing only few samples (Figures 4a and 4b), indicating that it detects data sets with similar distributions. However, in our experiments, the implementation was changed to ignore the stopping criterion and run continuously to inspect the subsequent behavior. The AUROC (Figure 4a) bounces around 0.49, but does not deviate much. The relative bias changes only slightly at the end (Figure 4c). With few exceptions, the representative ratio is retained until the data set is too small.

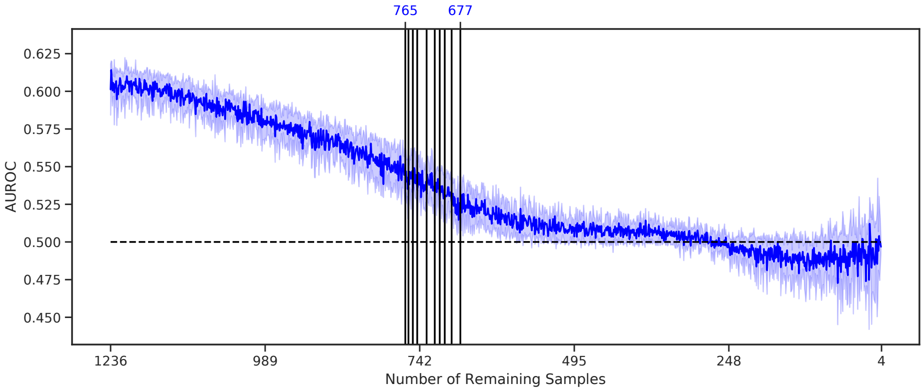

(a) AUROC

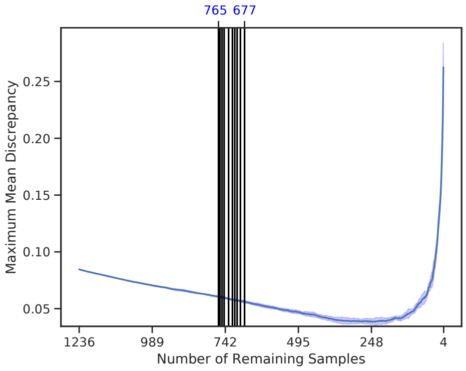

(b) MMD

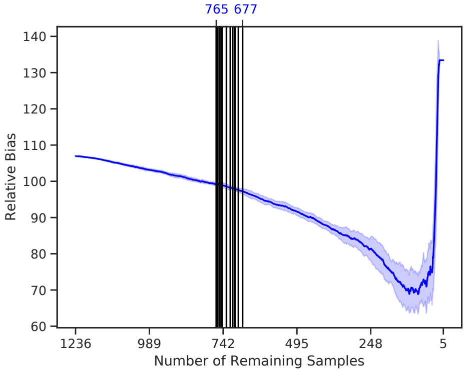

(c) Relative bias for income.

Supplementary Figure 3: AUROC, ROC curve, and MMD of the statistical correction of the US Census Income subset containing less positive samples (high income) with the auxiliary information of the representative subset. The vertical lines indicate the iteration in which the remaining samples were declared to be maximal representative subsamples, and the top numbers represent the remaining samples.

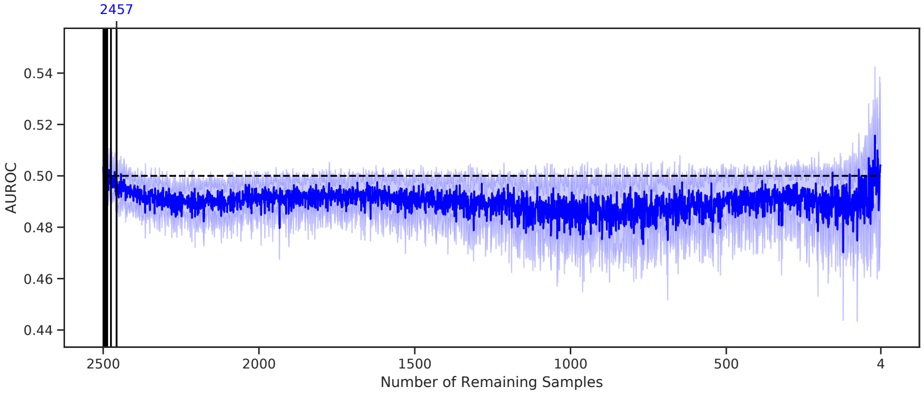

(a) AUROC

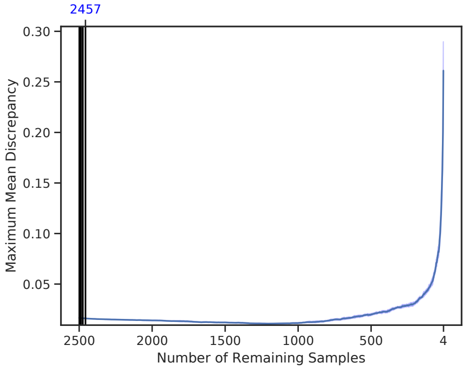

(b) MMD

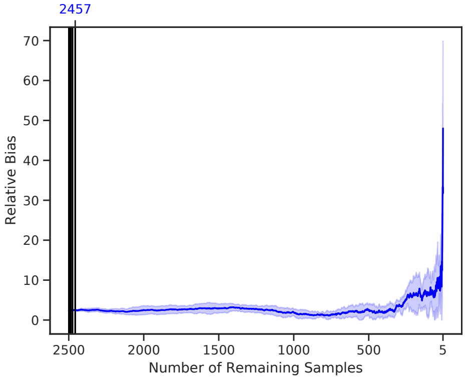

(c) Relative bias for income

Supplementary Figure 4: AUROC, ROC curve, and MMD of the statistical correction of the second representative subsample of income from the US Census (high income) with the auxiliary information of the representative subset. The vertical lines indicate the iteration in which the remaining samples were declared to be maximal representative subsamples, and the top numbers represent the remaining samples.

C Method Comparison

Supplementary Table 1: Downstream task MMD over 50 iterations. The numbers are mean values and standard deviations. The mean rankings over the data sets were once computed for all data sets and once for the three data sets with more than 2000 samples (US Census Income, US Census Employment and Human Research Analytic). Best values are written bold and second best italic.

Method	US Census Income	US Census Employment	Human Research Analytic	Breast Cancer	Loan	Mean Rank	Large Mean Rank
Uniform	0.056 ± 0.005	0.045 ± 0.004	0.052 ± 0.004	0.070 ± 0.011	0.098 ± 0.027	4.8	4.67
PSA	*0.042 ± 0.004*	0.025 ± 0.002	0.032 ± 0.006	0.040 ± 0.005	0.049 ± 0.008	2.8	2.67
KMM	0.043 ± 0.004	*0.023 ± 0.002*	*0.029 ± 0.006*	**0.023 ± 0.004**	**0.032 ± 0.006**	*1.8*	*2.33*
MRS	0.056 ± 0.004	0.045 ± 0.004	0.050 ± 0.005	0.065 ± 0.011	0.093 ± 0.025	4.2	4.33
Soft-MRS	**0.032 ± 0.003**	**0.019 ± 0.002**	**0.027 ± 0.005**	*0.029 ± 0.004*	*0.033 ± 0.005*	**1.4**	**1.00**

Supplementary Table 2: Downstream task relative bias over 50 iterations. The numbers are mean values and standard deviations. The mean rankings over the data sets were once computed for all data sets and once for the three data sets with more than 2000 samples (US Census Income, US Census Employment and Human Research Analytic). Best values are written bold and second best italic.

Method	US Census Income	US Census Employment	Human Research Analytic	Breast Cancer	Loan	Mean Rank	Large Mean Rank
Uniform	6.434 ± 3.273	7.128 ± 3.313	5.635 ± 5.433	5.775 ± 4.952	11.975 ± 5.232	4.2	3.67
PSA	4.471 ± 2.709	4.157 ± 2.247	5.708 ± 4.661	*4.515 ± 3.433*	*2.379 ± 1.674*	*2.8*	3.33
KMM	*4.205 ± 2.988*	*3.414 ± 2.639*	6.476 ± 5.274	4.950 ± 3.863	2.681 ± 1.995	3.0	*3.00*
MRS	6.966 ± 3.179	7.679 ± 3.310	**5.119 ± 4.285**	5.518 ± 4.895	11.030 ± 4.840	3.8	3.67
Soft-MRS	**2.116 ± 1.701**	**2.332 ± 1.849**	*5.412 ± 4.772*	**3.860 ± 3.411**	**1.486 ± 1.062**	**1.2**	**1.33**

D Data Set Characteristics

Supplementary Table 3: Characteristics for the data sets used in the experiments. Gesis and Allensbach contain no target feature.

Name	#Samples	#Positive	#Negative	#Features
US Census Employment	378817	172803	206014	99
US Census Income	195665	85189	110476	69
Human Research Analytic	8955	1483	7472	33
Gesis	3869	$\emptyset$	$\emptyset$	40
Allensbach	1082	$\emptyset$	$\emptyset$	54
Breast Cancer	683	444	239	10
GBS	579	550	29	52
Loan	480	332	148	13
